# Artificial intelligence-based model for predicting pulmonary arterial hypertension on chest x-ray images

**DOI:** 10.1186/s12890-024-02891-4

**Published:** 2024-02-27

**Authors:** Shun Imai, Seiichiro Sakao, Jun Nagata, Akira Naito, Ayumi Sekine, Toshihiko Sugiura, Ayako Shigeta, Akira Nishiyama, Hajime Yokota, Norihiro Shimizu, Takeshi Sugawara, Toshiaki Nomi, Seiwa Honda, Keisuke Ogaki, Nobuhiro Tanabe, Takayuki Baba, Takuji Suzuki

**Affiliations:** 1 Department of Respirology, Graduate School of Medicine, Chiba University, Chiba, Japan; 2 Pulmonary Hypertension Center, Chibaken Saiseikai Narashino Hospital, Chiba, Japan; 3 Department of Pulmonary Medicine, School of Medicine, International University of Health and Welfare (IUHW), Chiba, Japan; 4 Department of Radiology, Tsudanuma Central General Hospital, Chiba, Japan; 5 Diagnostic Radiology and Radiation Oncology, Graduate School of Medicine, Chiba University, Chiba, Japan; 6 Maebara Shimizu Eye Clinic, Chiba, Japan; 7 Chiba University Hospital Translational Research and Development Center, Chiba, Japan; 8 M3 Inc., Tokyo, Japan; 9 Department of Ophthalmology and Visual Science, Graduate School of Medicine, Chiba University, Chiba, Japan

**Keywords:** Pulmonary arterial hypertension, Artificial intelligence, Deep learning, Chest X-ray

## Abstract

**Background:**

Pulmonary arterial hypertension is a serious medical condition. However, the condition is often misdiagnosed or a rather long delay occurs from symptom onset to diagnosis, associated with decreased 5-year survival. In this study, we developed and tested a deep-learning algorithm to detect pulmonary arterial hypertension using chest X-ray (CXR) images.

**Methods:**

From the image archive of Chiba University Hospital, 259 CXR images from 145 patients with pulmonary arterial hypertension and 260 CXR images from 260 control patients were identified; of which 418 were used for training and 101 were used for testing. Using the testing dataset for each image, the algorithm outputted a numerical value from 0 to 1 (the probability of the pulmonary arterial hypertension score). The training process employed a binary cross-entropy loss function with stochastic gradient descent optimization (learning rate parameter, α = 0.01). In addition, using the same testing dataset, the algorithm’s ability to identify pulmonary arterial hypertension was compared with that of experienced doctors.

**Results:**

The area under the curve (AUC) of the receiver operating characteristic curve for the detection ability of the algorithm was 0.988. Using an AUC threshold of 0.69, the sensitivity and specificity of the algorithm were 0.933 and 0.982, respectively. The AUC of the algorithm’s detection ability was superior to that of the doctors.

**Conclusion:**

The CXR image-derived deep-learning algorithm had superior pulmonary arterial hypertension detection capability compared with that of experienced doctors.

**Supplementary Information:**

The online version contains supplementary material available at 10.1186/s12890-024-02891-4.

## Background

Pulmonary arterial hypertension (PAH) is a subtype of pulmonary hypertension (PH) and is characterized by elevated pulmonary arterial pressure due to increased pulmonary vascular resistance [1]. Symptoms of PAH are non-specific but commonly include exertional dyspnea and fatigue [2]. The estimated prevalence of PAH is 48–55 per 1 million adults in economically developed countries, and the typical course of PAH without treatment is death from right-sided heart failure. Currently, selective pulmonary vasodilators that act via three different pathways are available for treating PAH, and clinicians recommend an initial combination therapy [3].

Despite the establishment of treatment algorithms and reduced mortality due to PAH, the number of patients in the intermediate- and high-risk groups remain high (risk stratification according to the European Society of Cardiology and European Respiratory Society guidelines) [4, 5].

A contributor to the number of patients at risk is diagnostic delay. Diagnostic delay often occurs due to overlooked characteristic imaging findings signifying the presence of PH and related clinical symptoms [6]. A 2013 study reported that the mean time from symptom onset to diagnosis was 47 ± 34 months [6]. Moreover, patients reported 5.3 ± 3.8 general practitioner visits before being seen at a PH center [6]. At symptom onset, 95% of patients were classified according to the World Health Organization Functional Class (WHO-FC) II; however, the distribution of WHO-FC II, III, and IV in the same patient group at diagnosis was 0%, 94%, and 6%, respectively, representing a significant underestimation of the condition.

A recent study by Khou et al. reported that the mean time from symptom onset to diagnosis was 2.5 ± 4.1 years, and a longer diagnostic interval was associated with decreased 5-year survival. Therefore, although the treatment options for PAH are advancing, delays in diagnosis have not improved [7]. General physicians need to suspect PAH on chest X-ray (CXR) images in the early stages of the disease and refer patients to a PH center as appropriate [8]. Computer-aided detection/diagnosis (CAD) supports the detection and diagnosis of abnormalities or diseases. It can help physicians detect suspicious lesions that are easily overlooked, leading to improved detection accuracy [9].

CAD algorithms on CXR to detect pneumonia [10], lung nodules [11], pulmonary tuberculosis [12], and interstitial lung diseases [13] have been developed using deep-learning methods. Kusunose et al. developed and tested a deep learning algorithm to predict elevated pulmonary arterial pressure in patients with suspected PH using CXR images. In their study, the area under the curve (AUC) of the artificial intelligence (AI) algorithm was 0.71, and the negative predictive value (NPV) of the AI algorithm for detecting PH was 95.0%, providing promising results [14]. However, a deep-learning algorithm for PAH has yet to be developed.

To address this need, we aimed to develop and test a deep learning algorithm using the CXR images of patients with PAH. We hypothesized that the algorithm could help physicians detect PAH and provide better detection capabilities compared with that of doctors’ opinion alone.

## Methods

### Study design

This retrospective study used data collected from patients at Chiba University Hospital to develop and test a deep-learning algorithm using CXR images. All study protocols were performed in accordance with the Declaration of Helsinki. The Research Ethics Committee of the Graduate School of Medicine, Chiba University approved this clinical study (approval number: 4203). All adult participants provided written informed consent to participate in this study. The study design followed the Checklist for Artificial Intelligence in Medical Imaging (CLAIM).

### Datasets

The dataset included 145 patients with PAH and 260 control patients, almost all residing in Tokyo or Chiba Prefecture and visited Chiba University Hospital between January 1, 2003, and December 31, 2020. Using a Swan-Ganz catheter, PAH was diagnosed based on a right heart catheter (RHC). The diagnosis of PAH was made using hemodynamic measurements according to the most recent World Symposium standards: mean pulmonary arterial pressure > 20 mmHg, pulmonary arterial wedge pressure ≤ 15 mmHg, and pulmonary vascular resistance > 3 wood units [2, 15].

Due to the rarity of PAH, one to three CXR scans were used independently during the clinical course of each of the 145 patients with PAH (259 total images). The RHC-CXR data pair from patients with PAH, performed within 3-day intervals, constituted the analysis dataset. The control group consisted of 260 patients who visited the Ophthalmology Department of Chiba University Hospital from January 1, 2015 to December 31, 2020 and had CXR images without suspicion of PH (260 total images). For the control group, we used CXR images which were confirmed to be free of PAH, obvious cardiac enlargement, or interstitial pneumonia by two respirologists.

### Development of a deep-learning method for PAH detection

The ResNet50 model pre-trained on ImageNet-1k was used for image classification. A fully connected layer was added to the final layer for binary classification [16]. A PyTorch framework was used for the ResNet50 model implementation [17]. The training was performed on a workstation using an NVIDIA Tesla T4 graphics processing unit (Santa Clara, CA, USA). All CXR datasets were split in a 4:1 ratio for development and testing. The developed CXR dataset was separated into training and validation subsets using the K-fold cross-validation method (K = 4). The patient identification number was used to separate the CXR images to ensure that multiple CXR images from the same patient were not distributed across the datasets. Before training, the image contrasts were normalized to an intensity range of 0–255 and resized to 320 pixels × 320 pixels. Following this, the neural network was trained using Stochastic Gradient Descent. The objective function was Binary Cross Entropy Loss and the learning rate equaled 0.001. Training was performed for 50 epochs. Data augmentation during training included left/right and up/down flipping, changes in saturation and hue, and cropping.

### Testing the detecting capability of the algorithm

The algorithm outputted a numerical value from 0 to 1 using the testing dataset (the probability of the PAH score) for each image. A receiver operating characteristic (ROC) curve was plotted to represent the sensitivity and false positive rate (1 - specificity) for every score cut-off, and the AUC was calculated. The optimal AUC threshold was determined using the Youden index and sensitivity and specificity were calculated.

### Comparing the performance of the algorithm versus the performance of the doctors

The detecting capability of the algorithm was compared with that of doctors (respirologists [> 8 years of experience], *n* = 7 and radiologists [> 14 years of experience], *n* = 2). The distance from the doctor’s eyes to the monitor was approximately 60 cm. The time allowance given to read each image was ≤ 5 s.

### Statistical analysis

We compared the ROC curve of this algorithm with that of the doctor’s reading results. All statistical analyses were performed with EZR (Saitama Medical Center, Jichi Medical University, Saitama, Japan), which is a graphical user interface for R (The R Foundation for Statistical Computing, Vienna, Austria). Statistical significance was set at *P* < 0.05.

## Results

### Clinical characteristics

Participants were divided into two groups; those with PAH (PAH group: 145 patients; mean age, 51.9 ± 16.1 years; 34 males) and the control (control group: 260 patients; mean age, 62.5 ± 9.6 years; 131 males). The characteristics of the patients with PAH included in this study are shown in Table 1.

**Table 1:** Baseline characteristics of the study population with PAH

Variable	Total population (*n* = 145)
Age	51.9 ± 16.1
Sex, n (%)	
Male	34 (23.4)
Female	111 (76.6)
IPAH, n (%)	53 (36.6)
HPAH, n (%)	9 (6.2)
Other comorbid conditions, n (%)	
Systemic sclerosis	26 (17.9)
Mixed connective tissue disease	9 (6.2)
Systemic lupus erythematosus	8 (5.5)
Rheumatoid arthritis	6 (4.1)
Dermatomyositis	1 (0.7)
Human immunodeficiency infection	1 (0.7)
Portal hypertension	11 (7.6)
Atrial septal defect	14 (9.7)
Ventricular septal defect	3 (2.1)
Partial anomalous pulmonary venous return	4 (2.8)
WHO functional class, n (%)	
Class I	6 (4.1)
Class II	53 (36.6)
Class III	69 (47.6)
Class IV	17 (11.7)
Right heart catheterization results	
Mean PAP, mmHg	41.4 ± 13.4
Mean PAWP, mmHg	8.4 ± 3.7
PVR, Wood unit	7.9 ± 5.5
CI, L/min/m^2^	3.2 ± 1.3

*Data are presented as number of patients (percentage), mean ± SD.

Abbreviations: IPAH: idiopathic pulmonary arterial hypertension; HPAH: heritable pulmonary arterial hypertension; WHO: World Health Organization; PAP: pulmonary artery pressure; PAWP: pulmonary arterial wedge pressure; PVR: pulmonary vascular resistance; CI: cardiac index

### Detection capability of the algorithm

**Fig. 1:**
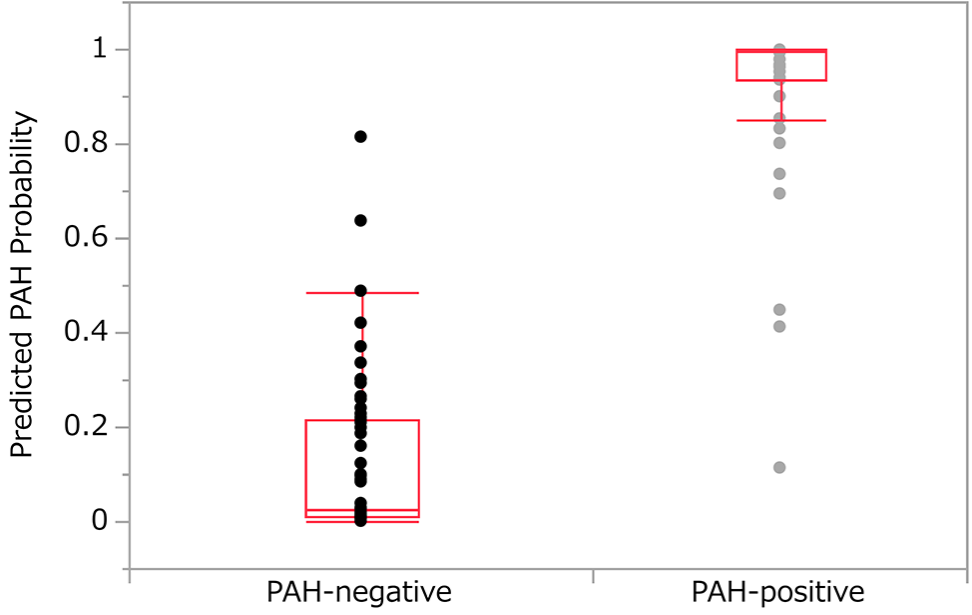
CXR images distribution. The figure shows the distribution of CXR images according to the algorithm-assigned score among PAH-negative and PAH-positive CRs included in the testing dataset. CXR: chest X-ray; PAH: pulmonary arterial hypertension

Figure 1 shows the distribution of CXR images included in the testing dataset according to the algorithm-assigned scores for those confirmed to be PAH-positive or PAH-negative.

**Fig. 2:**
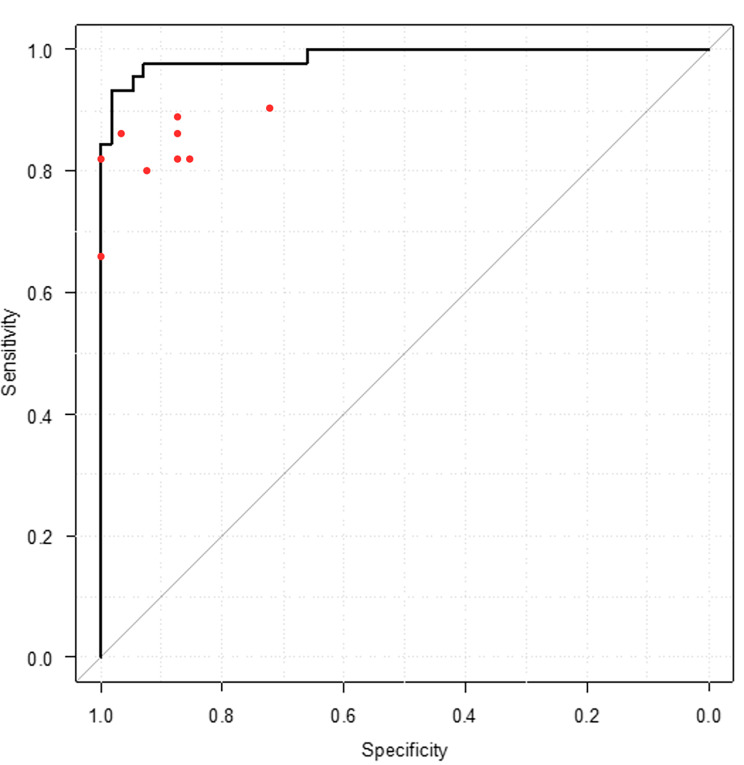
Diagnostic ability for pulmonary arterial hypertension. Comparison of the diagnostic sensitivity and specificity of the algorithm and the doctors’ opinion using images from the testing dataset. The red dots represent the doctor’s sensitivity and specificity. Black line: The ROC curve of AI algorithm ROC: receiver operating characteristic; AI: artificial intelligence

We divided the data sets into training and test sets, and then performed a four-fold cross-validation on the training set. The AUC of each model test on the test set was 0.9801 (SD, 0.0054). The final performance of the ensemble of four models was 0.988 (95% confidence interval [CI], 0.972–1.000). Using an AUC threshold of 0.69, the sensitivity and specificity of the algorithm were 0.933 and 0.982, respectively. Figure 2 shows the ROC curve of the AI algorithm using images from the testing dataset. In addition, all the doctors’ results are plotted. All plotted dots were below the ROC curve.

Additionally, AUCs were calculated for both sexes. The test dataset included 31 patients with PAH (PAH group: 9 males; 45 total images; mean age, 54.8 ± 5.6 years) and 56 patients without suspicion of PAH (control group: 30 males; 56 total images; mean age, 57.5 ± 8.0 years). There were no significant differences in age (*p* > 0.05). In the male-only group (39 males; 48 total images), the AUC of the AI algorithm for the detection of PAH was 0.993 (95% confidence interval [CI], 0.978–1.000). Using an AUC threshold of 0.801, the sensitivity and specificity of the algorithm were 0.967 and 0.944, respectively. Meanwhile, in the female-only group (48 females; 53 total images), the AUC of the AI algorithm for the detection of PAH was 0.983 (95% CI, 0.951–1.000). Using an AUC threshold of 0.693, the sensitivity and specificity of the algorithm were 1.000 and 0.926, respectively. Furthermore, the AUCs of the AI algorithm for both sexes were high.

### Performance of the algorithm versus the performance of the doctors

Among the nine doctors who interpreted the CXR images, the mean sensitivity and specificity were 0.829 and 0.906, respectively. The median (range) sensitivity and specificity were 0.822 (0.667–0.911) and 0.875 (0.821–0.982), respectively. For the detection of PAH, the AUC of the doctors was 0.945 (95% CI, 0.907–0.983), while that of the AI algorithm was 0.988 (95% CI, 0.972–1.000) and superior to that of the doctors (*p* = 0.0175). These findings demonstrated that the algorithm’s detection performance was superior to that of the doctors.

## Discussion

In this retrospective study, we developed and tested the CAD algorithms for analyzing CXR images to diagnose PAH. The AUC of the algorithm was 0.988, which was superior to that of experienced doctors. However, further real-world clinical trials are needed to determine how this algorithm can contribute to PAH detection.

CXR scans can be used as a simple and economical data source that are available globally. Recently, deep-learning AI diagnostic systems have been developed for chest imaging. Early detection and diagnosis of PAH are important because a longer diagnostic interval is associated with decreased 5-year survival [7]. If CAD improves the detection rate of PAHs on CXR images and makes this information available to non-specialist physicians, the time required to diagnose PAH can be reduced, which could lead to better prognosis.

**Fig. 3:**
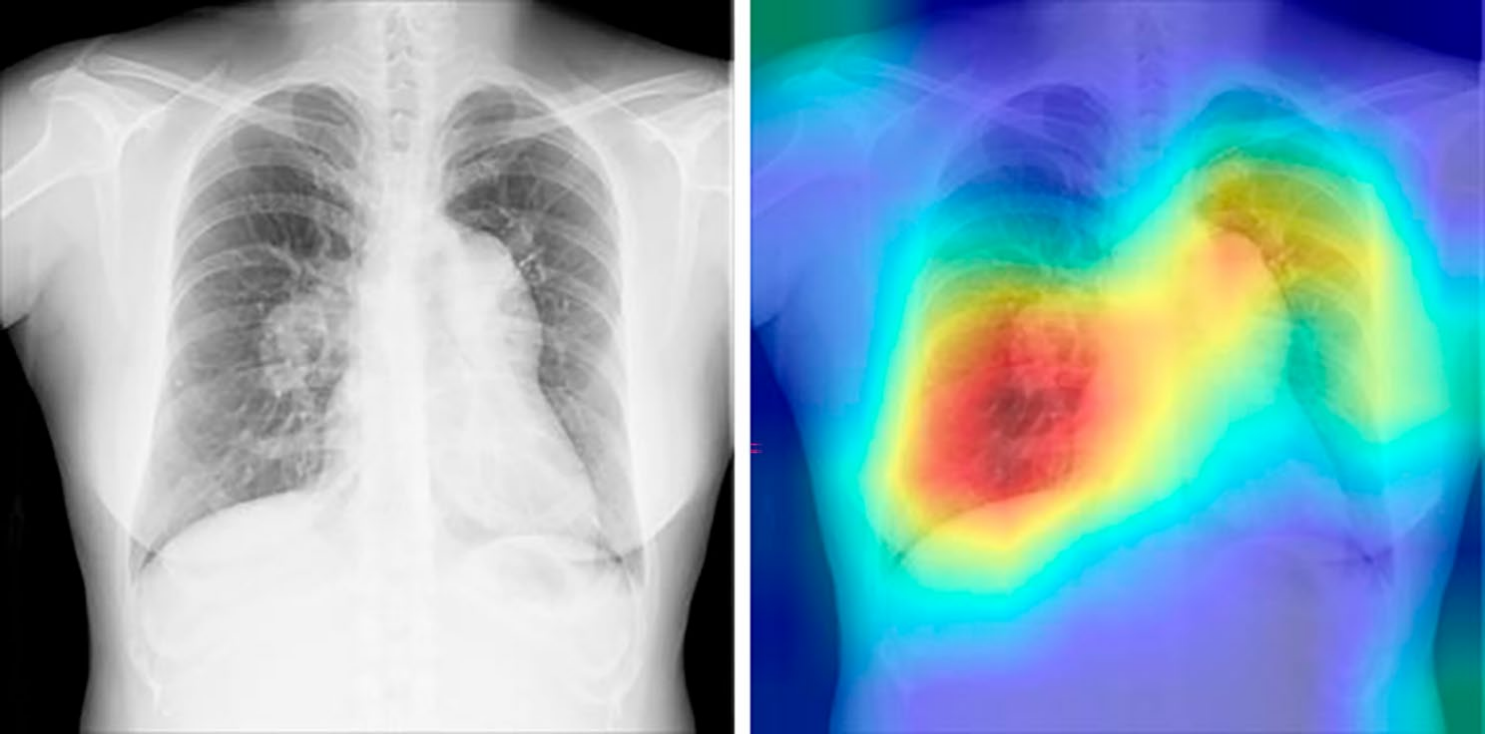
Analysis of the images where AI was focused. A single example demonstrating the function of the model. This CXR image was taken from a 39-year-old woman, with a mean pulmonary arterial pressure of 62 mmHg and WHO functional class III. Our AI model focused on the pericardial areas of the CXR images. AI: artificial intelligence; CXR: chest X-ray; WHO: World Health Organization

In this study, we developed an algorithm to extract suspected patients with PAH using CXR images and achieved an AUC of 0.988, albeit in a limited population. In a previous study, Kusunose et al. developed an algorithm to predict increased pulmonary arterial pressure using CXR images in a population with suspected PH, obtaining an AUC of 0.71 and a specificity of 0.95 [14]. However, the CAD task used was more difficult than that used in our study because the people in the control group (non-PH) had subjective symptoms and were suspected of having PH. To visualize the focus pixels of the model inference, we used Gradient-weighted Class Activation Mapping (Grad-CAM). This technique specifically allows for the visualization of the focus pixels per image. Figure 3 is a single example to demonstrate the function of the model. Our AI model focused on the pericardial areas of the CXR images (Fig. 3) and appeared to label for the presence or absence of PAH. Nine doctors diagnosed PAH on CXR images by mainly focusing on the enlargement of the pulmonary artery and the heart. The focused area of the AI model and the doctors suggests that the AI model has similar diagnostic criteria to those of the doctors. In cases of false-positive results in the control group, heart enlargement could be considered PAH by the AI algorithm (Supplementary Fig. 1). Whereas in the study by Kusunose et al., the AI program focused on the right upper lung area and the area around the heart because the right upper pulmonary field is generally a common site of focal congestion [14].

Future studies should include diseases with CXR images similar to PAH, such as cardiac hypertrophy, and collect data from diverse clinical settings. This study can serve as a precursor to allow for the development of algorithms for all PH groups. For example, validating the algorithm on various groups of patients in addition to those with PAH, such as those with chronic thromboembolic PH, would be beneficial. We believe this report will serve as a basis for future large-scale studies.

Our study had some limitations. First, the number of images used in the training dataset was relatively small because PAH is a rare disease. The small sample size could have impacted the accuracy of the algorithm as a larger training dataset would improve the algorithm’s performance. Therefore, further validation of the algorithm in a larger population in the future is required, including not only PAH but also PH patients with other diseases such as chronic thromboembolic PH. Second, CXR images were obtained from two forms of imaging equipment, including both imaging plates (IPs) and flat-panel detectors (FPDs). Computed radiography systems with FPDs generally have a higher image quality than those which use IPs. Therefore, differences in image quality and other factors may have affected the study results. In the future, consistency should be ensured regarding the type of imaging detector used. Third, due to ethical issues, RHC could not be performed in patients within the control group who presented with no symptoms of cardiovascular disease. Therefore, we could not completely discount PAH presence in the control group, indicating a potential validation bias in the participant selection strategy. However, as PAH is a very rare disease [3], we believe that it was not highly likely that patients in the control group had PAH. Fourth, the learning and testing protocols used for the algorithm were conducted at a single facility. Consequently, it is unclear whether the same performance can be achieved using CXR images under different conditions and with different equipment. Further clinical trials are required to validate this algorithm in a real-world setting.

In conclusion, we successfully developed a deep-learning algorithm which could detect PAH using CXR images. The detection capability of the algorithm was superior to that of experienced doctors. Analyzing CXR images with our algorithm could provide general physicians and non-specialists for PAH with an opportunity to suspect PH, which might lead to early diagnosis.

### Electronic supplementary material

Below is the link to the electronic supplementary material.

Supplementary Material 1

Supplementary Material 2

Supplementary Material 3

## Data Availability

The data supporting this study’s findings are available from the corresponding author, Shun Imai, upon reasonable request.

## Abbreviations

PAH: pulmonary arterial hypertension
PH: pulmonary hypertension
WHO-FC: World Health Organization Functional Class
CXRs: chest X-rays
CAD: computer-aided detection/diagnosis
AI: artificial intelligence
AUC: area under the curve
NPV: negative predictive value
RHC: right heart catheter
ROC: receiver operating characteristics
