# Supplementary figures and images for: Artificial intelligence-based model for predicting pulmonary arterial hypertension on chest x-ray images
Source: BMC Pulm Med. 2024 Feb 27;24:101. doi: 10.1186/s12890-024-02891-4 (PMC10898025; doi:10.1186/s12890-024-02891-4)

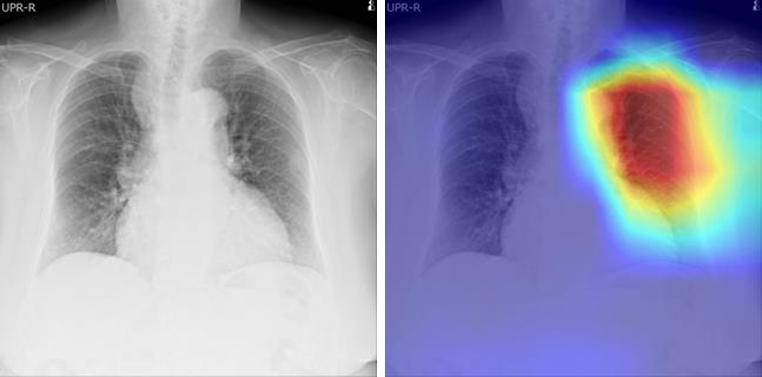

Supplement: Supplementary file 1 — Supplementary Material 1 [file 12890_2024_2891_MOESM1_ESM.tif]

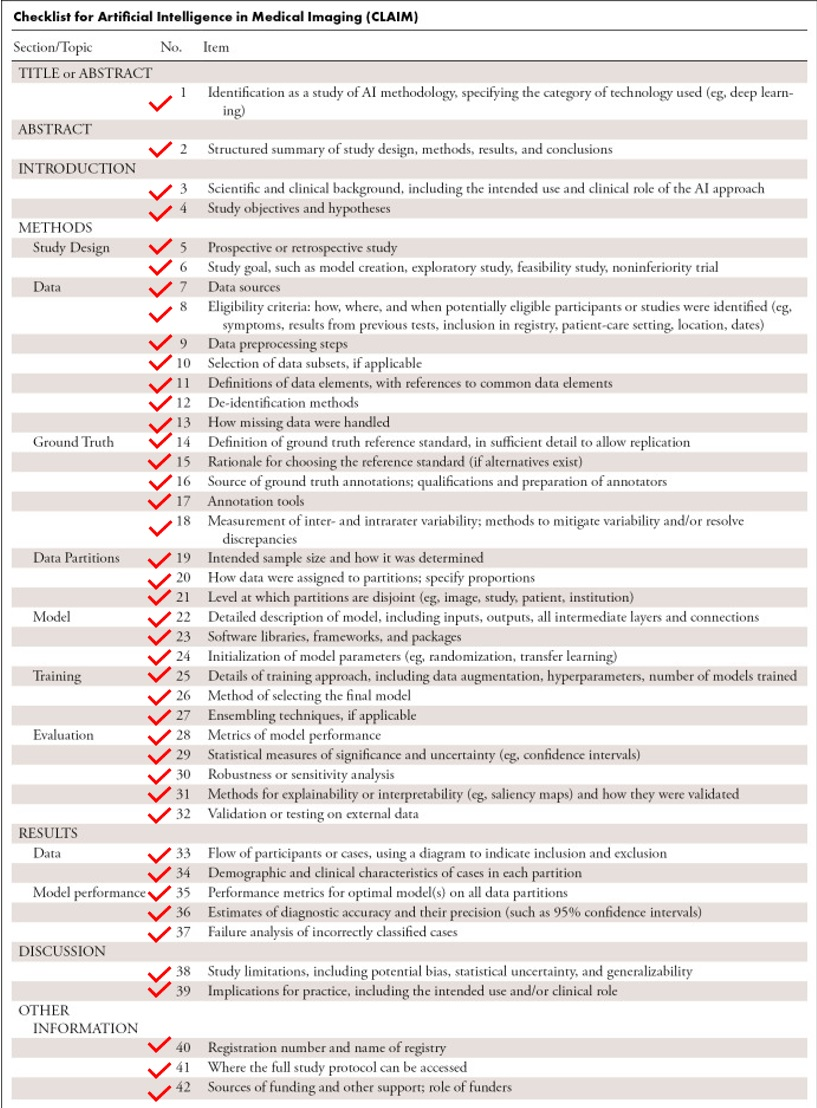

Supplement: Supplementary file 3 — Supplementary Material 3 [file 12890_2024_2891_MOESM3_ESM.tiff]
