# Supplementary material for: Artificial intelligence-based model for predicting pulmonary arterial hypertension on chest x-ray images
Source: BMC Pulm Med. 2024 Feb 27;24:101. doi: 10.1186/s12890-024-02891-4 (PMC10898025; doi:10.1186/s12890-024-02891-4)
Supplement: Supplementary file 2 — Supplementary Material 2 [file 12890_2024_2891_MOESM2_ESM.docx]

**SUPPLEMENTARY MATERIAL:**

Supplementary Figure 1. In cases of false-positive results in the control group, enlargement of the heart could be considered PAH by the AI algorithm. PAH, pulmonary arterial hypertension; AI, artificial intelligence.
